# Motor Learning Abilities Are Similar in Hemiplegic Cerebral Palsy Compared to Controls as Assessed by Adaptation to Unilateral Leg-Weighting during Gait: Part I

**DOI:** 10.3389/fnhum.2017.00049

**Published:** 2017-02-08

**Authors:** Diane L. Damiano, Christopher J. Stanley, Thomas C. Bulea, Hyung Soon Park

**Affiliations:** ^1^Functional and Applied Biomechanics Section, National Institutes of HealthBethesda, MD, USA; ^2^Mechanical Engineering Department, Korea Advanced Institute of Science and TechnologyDaejeon, South Korea

**Keywords:** asymmetry, aftereffects, cerebellar deficits, brain injury, children

## Abstract

**Introduction**: Individuals with cerebral palsy (CP) demonstrate high response variability to motor training insufficiently accounted for by age or severity. We propose here that differences in the inherent ability to learn new motor tasks may explain some of this variability. Damage to motor pathways involving the cerebellum, which may be a direct or indirect effect of the brain injury for many with CP, has been shown to adversely affect the ability to learn new motor tasks and may be a potential explanation. Classic adaptation paradigms that evaluate cerebellar integrity have been utilized to assess adaptation to gait perturbations in adults with stroke, traumatic brain injury and other neurological injuries but not in children with CP.

**Materials and Methods**: A case-control study of 10 participants with and 10 without hemiplegic CP within the age range of 5–20 years was conducted. Mean age of participants in the CP group was slightly but not significantly higher than controls. Step length and swing time adaptation, defined as gradual accommodation to a perturbation, and aftereffects, or maintenance of the accommodation upon removal of the perturbation, to unilateral leg weighing during treadmill gait were quantified to assess group differences in learning.

**Results**: Adaptation and aftereffects were demonstrated in step length across groups with no main effect for group. In CP, the dominant leg had a greater response when either leg was weighted. Swing time accommodated immediately (no adaptation) in the weighted leg only, with the non-dominant leg instead showing a more pronounced response in CP.

**Discussion**: This group of participants with unilateral CP did not demonstrate poorer learning or retention similar to reported results in adult stroke. Deficits, while not found here, may become evident in those with other etiologies or greater severity of CP. Our data further corroborate an observation from the stroke literature that repeated practice of exaggerating the asymmetry (error augmentation), in this case by weighting the more involved or shorter step leg, vs. minimizing it by weighting the less involved or longer step leg (error reduction) may be a useful training strategy to improve step symmetry in unilateral CP.

## Introduction

Human gait is a highly versatile process with continual adjustments to ever-changing external environments. These adjustments may be anticipatory (e.g., see an object up ahead) or reactive (e.g., surface is unexpectedly slippery) and may occur immediately or through an iterative process of error recognition and correction. Some particularly novel or challenging, as well as persistent, perturbations may lead to motor adaptation during functional tasks such as gait or reaching, which is the modification of a well-learned motor behavior through a process of adjustment to an altered task demand (Martin et al., 1996). Adaptation requires a period of practice with gradual accommodation, or incremental error reduction, to a new demand such that when it is removed, the new behavior will persist for a brief period. This transient persistence is referred to as an *aftereffect* and is evidence that the central nervous system has stored the adaptation at least temporarily (Martin et al., 1996; Bastian, 2008).

Studies have shown healthy adults (Savin et al., 2010) and even very young children (Musselman et al., 2011) can adapt to and temporarily store novel motor patterns within a single session. Most clinical studies have evaluated adaptation in adults with cerebellar damage (Ilg et al., 2008) who frequently present with motor learning deficits or those with unilateral brain injuries due to stroke (Reisman et al., 2010; Malone and Bastian, 2014). Split-belt treadmill paradigms have been utilized frequently to assess gait adaption and aftereffects within a single session (Reisman et al., 2007) and more recently as a repetitive training strategy to reduce step length asymmetry in adults post-stroke (Reisman et al., 2013). Investigators have been able to differentiate parameters that “adapted” or showed gradual accommodation such as step length, from those that had a more immediate response to the split-belt perturbation such as stance and swing times. The interpretation was that the latter did not require learning and consequently also did not demonstrate aftereffects (Reisman et al., 2005).

Investigators have hypothesized that with repetition (training), temporary learning during a single adaptation session could be reinforced and made to persist far longer. In one such training study, 12 subjects were post-stroke trained for 12 sessions over a 4-week period using the split-belt paradigm (Reisman et al., 2013). Counter-intuitively, subjects practiced walking with the belt speed slower on the side with the initially longer step length thus exaggerating the asymmetry. Slightly more than half (7/13) were deemed “responders” in that step symmetry post training that improved more than the individual difference between the two baseline sessions. However, nearly half did not improve suggesting that training using alternate strategies such as increasing, rather than slowing, the belt speed on the longer side, should also be investigated.

Responses to adaptation paradigms may vary not only due to brain pathology but also to maturation of brain pathways during normal development. Musselman et al. (2011) studied adaptation in very young children 3 years of age or less who were able to walk continuously on a treadmill using a split-belt paradigm to examine normal development of this phenomenon. Adaptation in step length was seen in 12 of 26 children. Authors postulated that inter-individual differences in the degree or rate of myelination of cerebellar tracts may explain developmental variations in adaptation.

Less commonly, other types of perturbations besides split treadmill belts have been utilized in adaptation paradigms with similar effects. These include unilateral leg weighting during treadmill walking which demonstrated both gradual accommodation in lower limb kinematics and a pronounced aftereffect (Noble and Prentice, 2006) or podokinetic stimulation (rotating treadmill) which produced consistent after-rotation during stationary stepping in healthy subjects that was shown to be disrupted in cerebellar patients (Earhart et al., 2002).

Cerebral palsy (CP), the focus of this study, encompasses a group of brain disorders occurring early in life with a resultant motor disability as the hallmark feature. Cerebellar abnormalities have recently been documented in unilateral CP and related to arm function (Fiori et al., 2015). The ability to adapt to a novel task demand and retain it may be predictive of the ability to learn or improve motor skill through training and therefore may prove to be an important source of the variance in response to training paradigms observed in this population. To our knowledge, no classic adaptation studies, in which subjects adjust to a new perturbation during a learned task such as reaching or gait and its subsequent removal, have been conducted in children with CP. The objective of this study was to compare how children with unilateral CP and without CP are able to accommodate to a novel gait perturbation and how well individuals with CP can temporarily retain new motor behaviors compared to those within the same age range but without CP. We hypothesized that, on a group level, children with unilateral CP would have poorer adaptation to the weighting of each leg compared to controls. We further hypothesized that they would have less pronounced aftereffects on weight removal, also indicating diminished learning capabilities. Gait adaptation paradigms that involve being subjected to two different treadmill belt speeds, or unilateral weighting, as done here, or bilateral weighting of the lower extremities (Vashista et al., 2013) while walking may also provide novel therapeutic approaches for improving step length or kinematic asymmetry in those with unilateral CP similar to studies in stroke, as well as addressing other types of gait deviations in those with bilateral involvement.

## Materials and Methods

### Participants

The recruitment goal was to obtain complete data sets on 10 participants less than 21 years of age with unilateral CP and 10 without CP of similar age to serve as a control, based on the sample size in a similar unilateral weighting paradigm in nondisabled adults (Savin et al., 2010) and in a second study examining adaptation in a children with unilateral brain injuries (Choi et al., 2009). All participants had to be at least 5 years of age to be able to comply well with instructions, and have had no surgery within the previous year and no leg injury besides CP that would affect their ability to walk. All were recruited and completed the study within a 2 year period. Participants with hemiplegia were self-referred or referred by local physicians or therapists, and controls were selected from a recruitment database based on similarity in gender and age to the clinical group. We further restricted the weight of all participants to 150 lbs maximum so that the percent of load (which we set at an absolute maximum of 12 lbs based on pilot testing) would not go below 8% of body weight. The study was approved by the institutional review board (Protocol #90-CC-0168). Written informed consent was obtained from participants 18 years and older. For those younger than 18, written consent was obtained from a parent or legal guardian as well as written assent of the participant. The setting was a motion analysis laboratory in a large research hospital.

Participants ranged from 5 to 20 years of age and included 10 with unilateral CP, mean age 14.8 ± 3.8 years and 10 controls without CP, mean age of 11.4 ± 3.6 years, with relevant group characteristics summarized in Table 1. Two additional participants with CP were enrolled in, but did not complete, the study. One needed to hold onto treadmill side rails when walking and the other was unable to lift the weighted leg well enough to sustain a consistent pace on the treadmill. These two participants were replaced to meet the recruitment goal. The rest of the participants completed the study with no reports or evidence of fatigue. Mean age and weight were slightly but not significantly higher in the group with CP.

**Table 1:** **Summary of demographic, physical and study-specific characteristics of participants**.

	**Unilateral CP (*n* = 10)**	**Control group (*n* = 10)**
Gender	6 M, 4 F	6 M, 4 F
Age (years)	14.8 ± 3.8	11.4 ± 3.6
Body mass (lb.)	115.8 ± 26.6	103.9 ± 34.5
GMFCS Level	I (3), II (7)	N/A
Side dominance	Right (3), Left (7)	Right (8), Left (2)
Treadmill speed (m/s)	0.93 ± 0.13	1.05 ± 0.13
Ankle weight (% BW)	8.72%	8.89%

**Figure 1:**
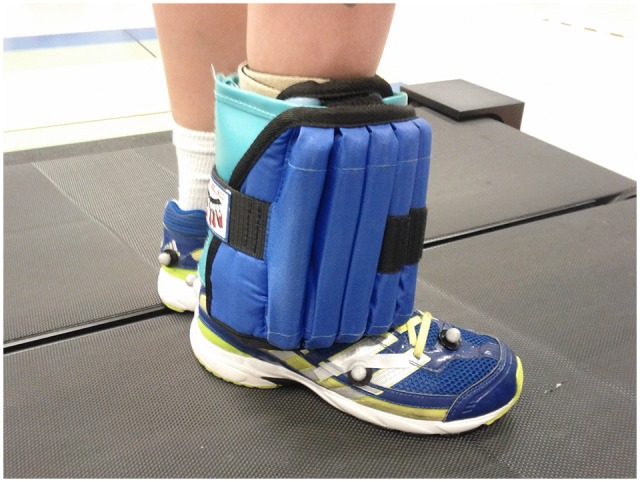
**Participant shown standing on the treadmill with the unilateral ankle weight attached**.

All with CP had a diagnosis of hemiplegia predominantly due to unilateral stroke (*n* = 7) with others due a bleed secondary to an arterial-venous malformation (1) tumor resection (1) and asymmetric periventricular leukomalacia (1) all of which occurred early in life. The actual ankle weights used were on average 8.72% and 8.89% of body weight for CP and control groups, respectively, due to a fixed limit to the maximum amount of weight (12 lbs). Figure 1 depicts the weight as placed on one of the subjects.

### Procedures

Prior to data collection, reflective markers were placed at specific locations to record lower extremity kinematics and temporal spatial data during the walking trials using a 3D motion capture system. Data were collected with Nexus (Vicon Motion Systems, Denver, CO, USA) and processed with Visual3D (C-Motion; Germantown, MD, USA) and Matlab (Mathworks, Natick, MA, USA). Only temporal-spatial data are reported here.

Each participant completed a single test session lasting less than 2 h which consisted of multiple treadmill walking trials (Bertec, Columbus, OH, USA) at self-selected speed as estimated from three trials of over ground walking. For all treadmill trials, participants wore a harness without weight support (Zero-G; Aretech, Ashburn, VA, USA) to ensure safety since they were instructed not to use the handrails. The gait adaptation paradigm consisted of a unilateral ankle weight of approximately 10% of body weight up to a maximum of 12 lbs which was deemed sufficient through preliminary testing to induce changes in the walking pattern without making it too difficult to walk. The weight was secured firmly onto the lower calf with Velcro with care not to place it so low as to interfere with ankle motion.

Each child performed five walking trials: a 2-min baseline, a 6-min trial with the weight on the non-dominant leg, a 2-min post-weight trial, a second 6-min trial with the weight on the dominant leg, and a final 2-min post-weight trial. A brief rest was provided at the midpoint of each 6-min weighted trial to reduce leg fatigue. Each trial began with the subject standing on the treadmill, then the belt accelerated at 0.3 m/s^2^ up to the self-selected speed. The weight was attached and removed between trials while the participant stood still on the treadmill. Participants were instructed not to move their feet before the post-weight trials. Motion capture data were collected for the duration of each trial condition. We focused on two parameters commonly reported in other gait adaptation studies, step length and swing time. Since standardized procedures were utilized across groups and the primary outcomes were from gait analyses, sources of biases during testing or data analyses were well-controlled.

### Data Analyses

Mean values for step length and swing time were computed within groups for each leg during the baseline, weighted and post-weight trials. To evaluate changes across conditions, groups and legs, a generalized linear mixed model was used with leg (dominant and non-dominant) and condition (baseline, non-dominant leg weighted, post non-dominant weight, dominant leg weighted, post dominant leg weight) as within subject factors and group (control and CP) as the between subject factor, with *post hoc* tests performed as indicated and *p-values* adjusted for the number of comparisons.

To better evaluate changes over time during the weighted conditions, we separated the data in the first and second half of that trial for the *post hoc* analyses. Since adaptation is a gradual process of adjustment to the perturbation, we expected to see a greater change in the second half vs. the first half of the weighted trial. In contrast, if the adjustment to the weight was immediate as indicated by a greater change between baseline and the first half of the weighted trial, this would mean that there was not progressive error reduction or adaptation. Similarly, aftereffects are a transient persistence of a learned behavior, so if these were present, we would not necessarily expect to see a difference between the second half of the weighted trial and the post-weight trial, but there should be a difference from the baseline trial.

Step length asymmetry is a consistent finding in those with unilateral CP, so we were also interested in how unilateral weighting of each leg would affect symmetry while the weight was on and after it was removed. The main goal here was to assess the effect of the perturbation, weighting one leg, before and after adaptation to it occurred and also at the end of the session to determine whether a return to baseline was seen. For this, we averaged step lengths over the first and last 5 steps of each condition. A symmetry index (SI) was computed as the ratio of dominant over non-dominant step lengths and compared across the two weighted and post weight conditions and groups. To evaluate changes in SI, a general linear mixed model was used with the condition as the within subject factor and group as the between subject factor.

## Results

### Step Length and Swing Time

**Figure 2:**
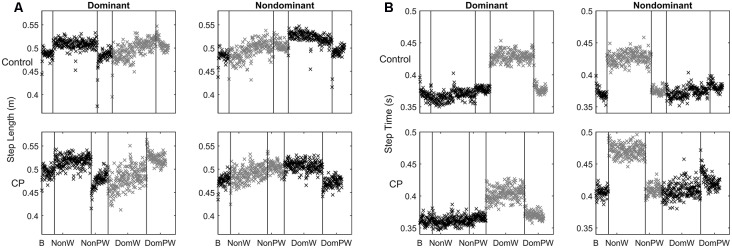
**(A,B)** Time series data for: **(A)** Step length shown here as the mean value for each step across each condition separated by group and leg with the weighted leg and its subsequent post-weight period shown in gray; and **(B)** Swing time shown here as the value for each step across each condition separated by group and leg with the weighted leg and its subsequent post-weight period shown in gray.

Figure 2A shows time series data for the averaged step length of each step per leg separated by group during the two unilateral weighted (dominant, non-dominant) and three unweighted conditions (baseline and two post-weight trials). Figure 2B shows similar data for swing time. Since the number of steps across subjects was not equal, the number of steps from the participant with the lowest number in each condition determined the number of steps included for averaging, with extra steps excluded always from the end of the each condition. Statistical results for mean differences across conditions by group, leg and parameter are shown in Table 2.

**Table 2:** **General linear mixed model results for step length, swing time and symmetry index with all *p*-values listed and those <0.05 indicated in bold**.

	Condition	Leg	Group	C × L	C × G	L × G	C × L × G
Step length	**<0.001**	0.68	0.89	**<0.001**	0.10	0.38	**0.04**
Swing time	**<0.001**	**<0.001**	0.16	**<0.001**	0.35	**<0.001**	0.10
Symmetry index	**<0.001**	–	0.38	–	**0.04**	–	–

*C, condition; L, leg; G, group*.

For step length, analyses showed a main effect for condition but no main effect for group or leg. The weighted conditions clearly altered behavior in both legs; however, the pattern of significant changes differed between the weighted and non-weighted leg as shown by the *post hoc* analyses for condition in Table 3 with results shown for each leg in the entire sample and also by group (with *p* values adjusted to <0.0031 for multiple [16] comparisons). In the unweighted legs, step length increased immediately as indicated by a significant increase from baseline to the first half of the weighted trials that was maintained but did not increase further in the second half of the weighted trials. Values also returned to those at baseline in the post-weight trials. In the weighted legs, the baseline condition did not differ significantly from the first half of either the weighted trials, but step length instead gradually increased during weighting. This lead to a significant difference from the first to the second half of the weighted period in the non-dominant weight condition that did not reach significance in the dominant weight condition even though the mean step length showed a similarly increasing trend. Step lengths failed to return to baseline and were significantly less than those of the post-weight trials for both weighted legs. These results indicate that only the weighted legs showed adaptation (i.e., more gradual increase) and persistence of after effects (i.e., no return to baseline). Since the perturbation was unilateral, it is not surprising that leg behavior varied depending on whether it was weighted or not; hence these results also explain why a significant interaction between condition and leg was found.

**Table 3:** ***Post hoc* comparisons for step length for each condition, separated by leg, in the entire sample and within groups with statistically significant changes indicated in BOLD (*p* value set at <0.0031 to account for multiple comparisons)**.

Comparisons	*p* value (all)	*p* value (Control)	*P* value (CP)
**NONDOM leg weighted**
Base-W1 NONDOM	0.970	0.926	0.966
W1-W2 NONDOM	**0.001**	**0.003**	0.123
W2-PW NONDOM	0.563	0.682	0.309
Base-PW NONDOM	**<0.001**	0.019	**0.002**
Base-W1 DOM	**0.001**	0.023	**<0.001**
W1-W2 DOM	0.287	0.635	0.367
W2-PW DOM	**<0.001**	0.006	**<0.001**
Base-PW DOM	0.509	0.423	0.116
**DOM leg weighted**
Base-W1 NONDOM	**<0.001**	**0.001**	0.009
W1-W2 NONDOM	0.131	0.072	0.822
W2-PW NONDOM	**<0.001**	**0.002**	**0.003**
Base-PW NONDOM	0.680	0.321	0.282
Base-W1 DOM	0.355	0.878	0.219
W1-W2 DOM	**0.001**	0.022	0.009
W2-PW DOM	0.066	0.629	0.026
Base-PW DOM	**<0.001**	0.022	**<0.001**

*NONDOM, non-dominant leg; DOM, dominant leg; CP, cerebral palsy; Base, baseline; W1, first half of the weighting period; W2, second half of the weighting period; PW, period after weight removal*.

The absence of a main effect for group indicates that children with CP had similar capabilities to children without CP in adapting their step lengths to a leg perturbation and in retention of the adaptation. As seen in Figure 2A, the sample with CP had greater variability in step lengths, as seen visually by more dispersion in the data points, than controls especially in the leg that was weighted, but their step length patterns were remarkably similar across groups. Aftereffects were also visually apparent in both post-weight conditions in both groups and were of similar magnitudes.

The lack of a main effect for leg seemed surprising at first since it is visually apparent that the legs behaved differently in CP vs. controls. However, this difference was revealed instead by a triple interaction between condition, leg and group. This can be explained by the tendency for children with unilateral CP to make greater adjustments with their dominant relative to their non-dominant leg regardless of which leg was weighted, which was not the case in the controls. *Post hoc* analyses for conditions within groups separated by leg, show this more clearly (Table 3). When the non-dominant leg was weighted, only the dominant leg in CP demonstrated a significant mean change from baseline to weighting and from weighting to weight removal. Differential group responses were also seen in the dominant weight condition. The control group showed a significant change from baseline to the first half of the dominant weight condition for the non-dominant leg that was not seen in CP, although both groups showed significant changes in the non-dominant leg from the second the half of the weighting trial to the post-weight condition. While not conclusive, these results further support the observed greater reliance on the dominant leg in the CP group.

For swing time, analyses revealed significant main effects for condition and leg, and leg by group and leg by condition interactions. No main effects for group were found. The main effect for leg is related to the immediate and persistent response on the weighted leg of decreased swing time. The leg by group interaction can be explained by asymmetry in the response magnitude across legs only in the CP group, with the non-dominant leg showing a more pronounced response to weighting in this case than the dominant leg. This contrasts the more similar response to weighting across legs in controls and is opposite to step length results in CP where the dominant leg made relatively greater adjustments.

The pattern for swing time while clearly distinct from that for step length, was again remarkably similar across groups. The weighted leg had a more apparent adjustment to the weight and it was immediate and consistent over the time period the weight was on (i.e., no adaptation). The post-weight response was a similarly strong and immediate return to baseline, rather than a brief persistence of the learned behavior (i.e., no after effects). The *post hoc* analyses supported this, revealing a significant mean change from the baseline to the weighted condition and from that to the post-weight condition for the weighted leg in both groups with no difference between the baseline and post-weight condition. Additionally, in the control group only, there was a significant mean change from the weighted to the post-weight trial in the leg opposite the one that was weighted.

**Figure 3:**
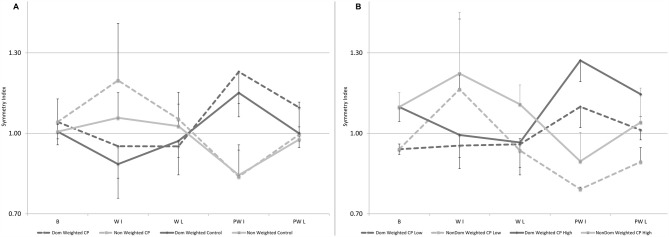
**(A,B)** Mean data for Symmetry Index (SI), defined as the ratio of dominant over non-dominant step length, shown here for baseline, and for the first and last five steps in the weighted and post-weight periods in: **(A)** the control group and the group with CP with the dominant leg weighted condition in black (or black-dotted) and the non-dominant weighted condition in gray (or gray-dotted). Errors bars are shown in one direction to avoid overlap and are directed up for the non-dominant leg and down for the dominant leg; and **(B)** the two subgroups with CP with the one having SI greater than normal (*n* = 6) shown by solid lines and the one having SI lower than normal (*n* = 3) in the dotted lines, with dominant leg weighted conditions in black for the subgroup with the high SI or black-dotted for the subgroup with the low SI and the non-dominant weighted condition in gray for the subgroup with the high SI or gray-dotted for the subgroup with the low SI. Error bars are shown in one direction only, this time by sub-group, with those for the solid lines pointing up and those with the dotted lines pointing down. Some error bars were too small to be visible beyond the data point.

Results for the SI for step length showed a main effect for condition. Basically when examining the group as a whole, with initial weighting the unweighted leg took longer steps lengths significantly increasing the asymmetry from baseline. Upon weight removal, there was a significant “rebound” effect in the opposite direction. Each of these reverted back towards baseline over time. No main effect for group was identified; however, a significant interaction effect between group and condition was found which likely reflected the asymmetry in the responses to weighting seen across legs in CP. Of the ten participants with CP, six had an initial SI more than one standard deviation greater than that in controls indicating a longer step length on the dominant side and three had a SI more than one standard deviation less than controls with one within the normal range. The pattern of change in SI for the control group compared to the entire group with CP and the two sub-groups with CP (those with greater or lesser asymmetry than normal) are shown in Figures 3A,B, respectively. Figure 3A illustrates the greater tendency in CP to rely on the dominant leg as seen by the upper shift in nearly all comparable data points. Figure 3B shows that in both subgroups with CP, error augmentation or weighting the leg with the shorter step length in CP, regardless of whether that leg is the dominant or non-dominant one, results in a greater limb symmetry at the end of the session.

## Discussion

Contrary to our hypothesis, those with unilateral CP did not differ from controls in ability to adapt their step lengths to a unilateral perturbation and to retain this adaptation. Masia et al. (2011) noted that children may have a less well-calibrated sensorimotor system than adults due to less motor experience and continual readjustments in response to rapid growth which makes learning less efficient for them. Children with CP may have even greater motor “noise” as seen here by their more variable responses to weighting than those without CP; however, it is interesting to note that their learning abilities were not found to differ from controls.

Similar findings of no differences in adaptation have interestingly been reported in adults post-stroke and with Parkinson Disease and authors attributed these results to little if any involvement of the cerebellum in those patient groups (Reisman et al., 2010). In contrast, patients with clear cerebellar involvement (Ilg et al., 2008) and even those with more diffuse brain injuries such as traumatic brain injury (Vasudevan et al., 2014) did demonstrate impaired adaptation. In our sample, most had focal unilateral lesions (9 of 10), with seven of those having stroke as the primary etiology so it is perhaps not surprising that their response was similar to that in adult-onset stroke. A previous study comparing balance in children with and without CP, showed that the relative difference in performance between eyes open and closed balance conditions was similar across groups indicating no evidence of significant cerebellar involvement (Damiano et al., 2013). Children with CP in that study also had mild motor deficits and could stand independently although they had poorer balance than controls in the static eyes open position. Many with CP, especially those with bilateral brain injuries, tend to have more diffuse white matter (periventricular leukomalacia) or gray matter (anoxic) brain injuries, so they may be more likely to have direct or indirect involvement of the cerebellar pathways and therefore a greater likelihood of demonstrating deficits in motor learning than the children with unilateral CP who were studied here.

As seen in adults with stroke, children with unilateral brain injuries also demonstrate gait asymmetry that could perhaps be targeted using adaptation-based training approaches. Regnaux et al. (2008) had subjects with stroke undergo a single session where their unaffected leg was “constrained” using a unilateral weight on that side. Immediately after weighting and up to 20 min post, they observed a significant mean increase in loading on the affected side and increased gait speed. Individual step lengths were not reported but mean step length was 0.07 meters longer on the paretic side at onset. Both legs increased mean step lengths by the same amount immediately after training although only the change on the paretic side reached significance. After 20 min both increased again but the degree of step asymmetry remained virtually the same (mean paretic step length was 0.07 and 0.06 m longer immediately and after 20 min, respectively). Reisman et al. (2013) employed a similar strategy although the stated rationale was different: to exaggerate the asymmetry rather than constraining the better limb. Using a split-belt paradigm, they slowed belt speed on the side with the initially shorter step length which was the non-paretic side in 11/12 subjects and trained them this way for 12 sessions. Their subjects were categorized as either “responders” (*n* = 7) whose step asymmetry was reduced though bilateral increases in step length that were more pronounced on the initially shorter side, and “non-responders” (*n* = 5) whose step asymmetry did not change. Stance time asymmetry, although clearly manipulated here, did not change significantly in either subgroup.

While not reported, it is very possible that the mean results for their entire sample (responders plus non-responders; Reisman et al., 2013) failed to show a significant change in asymmetry from training, similar to the mean results from the previous single training bout study (Reisman et al., 2007); however, their study points to the importance of going beyond mean results and examining individual or sub-group responses to training to identify and ultimately characterize those that might benefit from a specific strategy (Damiano, 2014). In our study, the effects of loading *both* legs were examined so we could compare changes in asymmetry in response to each. As seen in Figure 3A, the control group showed a very symmetrical pattern across legs with a clear asymmetry in the response across legs in CP (note: only performance of the weighted leg is shown in this figure). Although sub-groups were too small for statistical analyses, in Figure 3B, we show the SI data separately for the participants with CP who have a longer step length on the dominant side (solid lines indicate SI is greater than 1; *n* = 6) vs. on the non-dominant side (dotted lines indicate SI less than 1; *n* = 3), with the one participant whose step asymmetry was within 1 standard deviation excluded. Our data indicate, similar to the conclusions from the two stroke studies, that loading (or speeding up in the split belt treadmill studies) the leg with the initially shorter step length leads to greater step symmetry in the retention phase, both immediately upon weight removal and at the end of the 2 min post-weight trial, and this principle holds for both sub-groups; whereas the opposite strategy on both sides failed to reduce and in some cases worsened the degree of asymmetry. Thus this study provides additional evidence that error augmentation may be more effective than reduction for improving step asymmetry.

Vaswani and Shadmehr (2013) further explored the important role of error in adaptation-induced change showing that as errors were artificially eliminated, the motor changes (learning) started to decay. They also postulated that adaptation involves two types of memories, one that disengages when the brain detects a change in the task and one that persists despite the change which they referred to as a trace of the motor memory. This may help explain why even after single session paradigm where the behavior rapidly shifts towards baseline on removal of the perturbation, there also appears to be some residual short term effect, as seen here in the improvement in symmetry. These findings have clinical relevance for therapists who have long recognized that they could perturb the system to facilitate a change in behavior within a treatment session. However, we now know that for these changes in behavior to persist, extensive practice is required. Also relevant to practice, therapists often try to incrementally improve or shape motor skill through practice, as seen for example in constraint-induced movement therapy approaches. However, stimuli that produce adaptation are typically fairly dramatic manipulations and may be an effective alternative strategy to shift motor behaviors that warrants further investigation.

Another interesting result here was that the leg asymmetry differed in the two parameters with the weighted non-dominant leg showing the greater relative change in swing time and the dominant leg showing slightly larger and more variable responses in step length when either leg was weighted. It could perhaps be concluded that individuals with CP rely more on the dominant leg to adjust to challenging perturbations simply because it has greater motor capabilities because cortical areas subserving them were not damaged. Studies in stroke have demonstrated greater connectivity in the unaffected vs. affected hemisphere and its dominant role particularly earlier in recovery (Bajaj et al., 2015). The change in swing time appears to be a more automatic or already well-learned response to the perturbation that does not involve further learning and may be stronger on the non-dominant side because the dominant side is more engaged in learning to accommodate step length to the perturbation. In the Musselman et al. (2011) study on infants and adults using the split-belt paradigm, they also showed immediate changes in stance time in all infants, regardless of their response to step length, suggesting that these had different neural mechanisms. Interestingly, they also showed that infants had slower rates of learning than adults, similar to the findings by Masia et al. (2011) in older children, and that the larger the error they experienced to the perturbation, the slower the learning.

One limitation of this study was the decision to weight the dominant leg first which we presumed would be easier and then proceed to the harder task of weighting the non-dominant leg instead of counterbalancing to avoid the possibility of order effects. However, we do not think that this had a significant effect on the results because the main comparison was between groups, each of which followed the same order and the response to unweighing was similar across legs in the control group. Another potential limitation was the possibility of fatigue especially in the children with CP. However, the values for step length and swing time in the final post-weight trials were either greater than or equal to the baseline values in both groups, indicating that fatigue was unlikely an issue here. Also, while the mean age across groups was not statistically different, the control group was several years younger than the group with CP. Since adaptation seems to increase with age, it is possible that older children with CP, while similar in adaptation ability to younger children without CP, may not be as capable as children of the exact same age.

In conclusion, in this first gait adaption study in CP, participants with unilateral CP did not differ in adaptation or retention from age-matched controls, suggesting similar abilities to learn new motor skills. Further examination of individual participants in this study or of children with different types of CP may be warranted to help explore as yet unexplained variability in responses to motor training that have been reported in CP. Weighting the side with shorter step length side, but not the converse, lead to improvements in step symmetry in CP as has been shown in stroke, suggesting that error augmentation may be an effective training strategy.

## Author Contributions

DLD, CJS, TCB and HSP had substantial contributions to the conception or design of the work; or the acquisition, analysis, or interpretation of data for the work; drafting the work or revising it critically for important intellectual content and final approval of the version to be submitted for publication. All (DLD, CJS, TCB, HSP) further agree to be accountable for all aspects of the work in ensuring that questions related to the accuracy or integrity of any part of the work are appropriately investigated and resolved.

## Funding

This work was funded by the Intramural Research Program of the NIH Clinical Center.

## Conflict of Interest Statement

The authors declare that the research was conducted in the absence of any commercial or financial relationships that could be construed as a potential conflict of interest.
